# egoDetect: Visual Detection and Exploration of Anomaly in Social Communication Network

**DOI:** 10.3390/s20205895

**Published:** 2020-10-18

**Authors:** Jiansu Pu, Jingwen Zhang, Hui Shao, Tingting Zhang, Yunbo Rao

**Affiliations:** 1 Visual Analytic of Big Data Lab, School of Computer Science and Engineering, University of Electronic Science and Technology of China, Chengdu 610054, China; 15928603008@163.com (J.Z.); sophyond@163.com (H.S.); tingting_uestc@163.com (T.Z.); 2 School of Information and Software Engineering, University of Electronic Science and Technology of China, Chengdu 610054, China; uestc2008@126.com

**Keywords:** anomaly detection, visualization, social communication, egocentric network, internet of things

## Abstract

The development of the Internet has made social communication increasingly important for maintaining relationships between people. However, advertising and fraud are also growing incredibly fast and seriously affect our daily life, e.g., leading to money and time losses, trash information, and privacy problems. Therefore, it is very important to detect anomalies in social networks. However, existing anomaly detection methods cannot guarantee the correct rate. Besides, due to the lack of labeled data, we also cannot use the detection results directly. In other words, we still need human analysts in the loop to provide enough judgment for decision making. To help experts analyze and explore the results of anomaly detection in social networks more objectively and effectively, we propose a novel visualization system, egoDetect, which can detect the anomalies in social communication networks efficiently. Based on the unsupervised anomaly detection method, the system can detect the anomaly without training and get the overview quickly. Then we explore an ego’s topology and the relationship between egos and alters by designing a novel glyph based on the egocentric network. Besides, it also provides rich interactions for experts to quickly navigate to the interested users for further exploration. We use an actual call dataset provided by an operator to evaluate our system. The result proves that our proposed system is effective in the anomaly detection of social networks.

## 1. Introduction

Social communication is a necessary part of people’s daily life. However, it seems that we suffer from all kinds of harassment every day, like sales phone calls, robots, harassment on social platform and so on. These seriously affect our daily life. Therefore, this has led to the development of anomaly detection. To best of our knowledge, the majority of the anomaly detection methods can be divided into two categories: unsupervised [1] and supervised [2] methods. However, there are still many challenges in anomaly detection. Firstly, the valid and objective tag data is hard to gain [3]. If we collect them all manually, it will take a long time and make the data more subjective and likely to lose their meaning. The number of anomalies is usually small and anomalies come in a variety of forms [3]. Besides, in practical use, we can only infer from the behavior or some characteristics of a user in the network without knowing exactly whether he or she is an abnormal user or not, which poses a greater challenge to the accuracy of anomaly detection.

Social network is a durable research topic, and theories for analyzing social networks are also emerging, such as the structural hole theory [4], egocentric network and Dunbar’s number [5]. Dunbar and Zhou discovered through research that an ordinary person’s social network is hierarchical [6,7], which means that if a person lacks this hierarchical structure, it is very likely that he or she is an anomalous user. In addition, the egocentric network allows experts to start by the topology and have an intuitive understanding of the user’s network, which is very helpful in identifying anomalies in social networks [8].

Visualization has a huge impact on evaluating data analysis results and mining data, and can help provide additional evidence to support ideas and conclusions. It also allows users to access the information and discover hidden connections in the data quickly by mapping the data into recognizable graphics. Therefore, it has been widely used in various fields, such as anomaly detection [9,10,11], social analysis [12,13,14] and so on. However, to design a general and effective visualization is a difficult problem, especially for anomaly detection in social networks. As the types of social network data are diverse, containing text, audio files, and video files, etc, it is hard to design a suitable model to cover all of them.

Combining the above questions and thoughts, we design a novel visualization system, egoDetect, which can explore anomalies from both global and local perspectives, and then, combine the time series to analyze users’ anomalies from multiple perspectives. It can detect anomalies in the data of social networks without tags. egoDetect based on ego central network provides three views for exploring and analyzing suspicious users. (1) A macroscopic view using the features of egos, analyzes all the egos’ degree of anomaly and displays from a group level. (2) Inspired by the solar, we propose a mesoscopic view to explore the nodes we are interested in. We can learn the topology of egos with alters and the characteristics of them from an ego central network perspective. (3) We also provide a microscopic view to reveal the behavior patterns and hobbies of the ego and the detail of the ego with a specific alter. In summary, our system can analyze users from three levels from multi-perspective. We also add friendly and intuitive interactions to help experts quickly get the information they want.

Our contributions in this paper as follows:

- We provide a novel visualization system for anomaly detection of social communication data, especially the unlabeled data. It combines anomaly detection algorithm with sociological theory, and then uses time sequence together for validation.
- Inspired by the solar system and the social brain hypothesis [5], we design a novel glyph to explore an ego’s topology and the relationship between egos and alters.
- We use a call record data provided by an operator to demonstrate the effectiveness of our system.

## 2. Related Work

### 2.1. Anomaly Detection

Anomaly detection is to identify points which are significantly different from other data [3,15]. For example, in the field of social analysis [16,17,18], anomalies refer to users with anomalous behaviors compared to the general public. They may be robots or highly active anomalous users [19]. Its core is how to identify the real anomalous points and avoid the wrong partition. With the input of data, we can get the results, like scores or labels. It is a very important issue in various fields, and many methods and tools are proposed. One of them is supervised machine learning methods [2,20], using tag data training model to classify the data. Another one is unsupervised machine learning methods [1,21,22], with no training data and thus has been widely used. H. Shao et al. use multi-modal microblog content features with analysis of propagation patterns to determine veracity of microblog observations [22]. However, because of the lack of an objective evaluation system, both of them is hard to evaluate. Therefore, an increasing number of experts and scholars tend to apply visualization analysis to anomaly detection. Histogram visualization is the most mature and popular method [23] due to its easy and intuitive to use. Especially in fraud detection and denning, their behavior is easy to capture and model into histogram [24,25]. Thom et al. design a visualization system for detecting anomalies based on label cloud [10]. N. Cao et al. propose a visualization system detecting anomalous users via Twitter [8]. Our system compares to them, based on ego central network to analyze the relationship between egos and alters, using a novelty design to explore users’ behavior effectively. The LOF algorithm we use can quantify the user’s anomalies into scores rather than just a single label, which is very helpful for the follow-up work. Besides, our system can be used in all social communication records.

### 2.2. Social Network Visualization and Analysis

With the analysis of social network, we can know individuals, groups or the whole network in a more effective way [12,26]. There are many kinds of topics in social network research, such as character recognition [27], information diffusion research [22,28,29], group detection [30], etc. Z. Qin et al. use homophily to increase the diffusion accuracy in social network [22]. J. Gao et al. find that the volatility of weak ties is very important for a person to make decisions and information diffusion [29]. Visualization methods are used extensively in social networks to enable intuitive research on abstract networks [31]. Zbigniew Tarapata et al. consider applying multicriteria weighted graphs similarity (MWGSP) method to examine some properties of social networks [32]. Vincent D Blondel et al. survey the contributions made so far on the social networks and explore large-scale anonymized datasets [33]. Jian Zhao et al. incorporate machine learning algorithms to detect anomalies and present an interactive visual analysis system called FluxFlow, which also offers visualization designs for presenting the detected threads for deeper analysis [34]. Based on node link network, J.Heer et al. design a system to explore the large graph structures using visualization [35]. Nardi et al. utilize colors to distinguish the communities that exist in users’ email contacts [36]. Mutton’s PieSpy gives us the opportunity to research the real-time dynamic community visualization in Internet-based chat systems [37].

However, all of the above studies are focused on a specific social network, which causes to a result that one research can only be used for one purpose, and does not have good scalability and generality. As is stated above, with the development of the Internet, there have been various social communication platform, which also leads to the complexity and heterogeneity of social data. N. Cao et al. proposed an initiator-centric model and a responder-centric model to tackle this problem [38]. Based on their study, we design an ego centric based model to crush this challenge.

### 2.3. Ego Centric Network Visualization and Analysis

Ego centric network analysis has been widely applied in anthropology and sociology. In the method, it assumes that one node called ego is in the center, and some nodes around it called alters. From the ego centric network, we can have a deeper understanding of interested nodes, obtaining the ego’s behavior and the structure with alters [39,40]. Mesoscopic and microscopic perspectives are two common starting points in it [4]. On the one hand, many researchers study the network structure and attributes of one or part of egos from a mesoscopic perspective. It is found that network size has a large effect on ego’s features and the composition of the network [41]. In Lubbers et al. research, they summarized that how long a relationship can be maintained depends mainly on the strength of the relationship, the density of the network [42]. On the other hand, the microscopic level focus on network properties, alters and their behaviors’ effect to the ego. L. Backstrom et al. found that the intimacy between couples can be indirectly determined based on the relationship between their mutual friends [19].

Nowadays, more and more researchers use advanced visualization methods to reveal more patterns in ego central network [43]. Shi et al. show the time dimension and the ego network’s structure by a 1.5D form [44]. Node-link model is the most commonly used visualization method in ego centered networks [4,45,46], where each vertex represents a person and each edge represents the strength of the relationship between two vertices. However, this method does not directly reflect the relationship between each alter and ego, and as the number of vertices increases, visual clutter will become serious. Different from the above studies, our system uses a novel glyph based on the solar, and is more intuitive to study the topology of the network and the relationship between egos and alters.

## 3. Problem Description

Our goal is to design a visual analytic system that can help detect, analyze and reason about why this person in the system is considered to be the anomalous user. In this section, we will define the problems that we need to solve.

### Problem Description

Generally, the data of social communication is noisy and huge. Typically we usually need to analyze on a real-time. Besides, experts with different backgrounds may have different ideas of whether a user in the network is anomalous or not. For example, Sociologists may judge whether a user is anomalous by the number of contacts he/she has; researchers of the algorithm may focus on calculating the similarity between the user and others in the network. Thus, we need to design a novel visualization system to help experts from different domains. Our goal is to find anomalies in unlabeled social data, which is difficult to analyze through only one perspective. For the follow-up study, we define the following questions.

- P1 Rate Ego’s Anomaly. Traditional anomaly detection only draws a conclusion of whether the point is an anomaly or not. Then here come problems: Are the two anomalous users having the same degree of anomaly? Is there something in common or in a difference of anomalous users. Therefore, we need to quantify a user’s degree of an anomaly and rank the users. Then experts can determine whether the user needs deep analysis.
- P2 Multi-perspectives Analysis. Since we know little about the unlabeled data, it easily leads to different people owning different results. In order to eliminate the influence of subjective factors, we need to design some indicators that can help us dissolve from multiple perspectives, such as the anomaly in the global social network and local topology, the time series of the user and alters anomalies.
- P3 Identify Anomalies. After we quantify the ego’s anomaly from multi-perspectives, the next thing we need to do is to identify the anomalies in the social networks. In our work, we mainly focus on those who have different behaviors from the general public. e.g., the number of contacts exceeds the Dunbar’s number, the topology of the user is strange, or the time series and behavior patterns are different from other people. We need to propose a novel visualization system to reveal the features and patterns of users and validate them, because they are the abstract of all types of social networks and play an important role in depicting the egos’ portraits.
- P4 Generality and Scalability. With the development of social communication, there are many kinds of social ways, such as Email, Twitter, Weibo and so on, which makes the data of them more and more complex and noisy. It is time-consuming and laborious if we design different systems for each type of social communication. Therefore, we want to design a more general model, which can be used in each type of social communication.
- P5 Rich Interaction. The main goal of our system is to help experts find out the anomaly in the social network full of unlabeled data, so it is necessary to have various interactions to help experts exploit the network by a custom way. For example, we need filtering and zooming for finding the ego we are interested in and looking for the detail about the network.

## 4. Detection in Social Networks

Anomaly usually refers to the part of users that behave differently from other users throughout the social networks. Thus, only by identifying those weird users can we make follow-up analysis and validation. In this section, we will introduce the egocentral based data model and the metrics used in detecting anomaly in our system. Then, we combine them into anomaly detection to find out the egos we need to explore deeply.

### 4.1. Data and Model

Communication data records the behaviors of how people communicate with each other and how they organize their social networks. For example, the Call Detail Records (CDRs) can be used not only to study the human communication behaviors but also to analyze Ego Networks (ENs). The call detail records are collected by mobile operators for billing and network traffic monitoring. The basic information of such data contains the anonymous IDs of callers and callees, time stamps, call durations, and so on.

In order to design a system using in all social networks, the first thing we need to do is to build a general model. However, this is not an easy task, because it not only needs to summarize from a wide variety of data, but also requires to make the features meet the requirements for anomaly detection. On the basis of existing research, we build a general model based on the egocentric network. The egocentric network can reveal the topology and features of egos and is useful in understanding the egos and validating them.

An egocentric network usually consists of a central node and several other nodes surrounding it, and there is a bond that allows egos and alters to connect with each other. For social networks, it is called contact. Contact is an important measure of the intimacy between egos and alters, as well as the structure of the egos’ network. Different social communication has different contact methods. For example, in the telecommunication or e-mail, it means I have a phone call or email with you, while in the Tweeter or Weibo, it means I retweet, comment or like under your tweet or vice versa.

**Figure 1:**
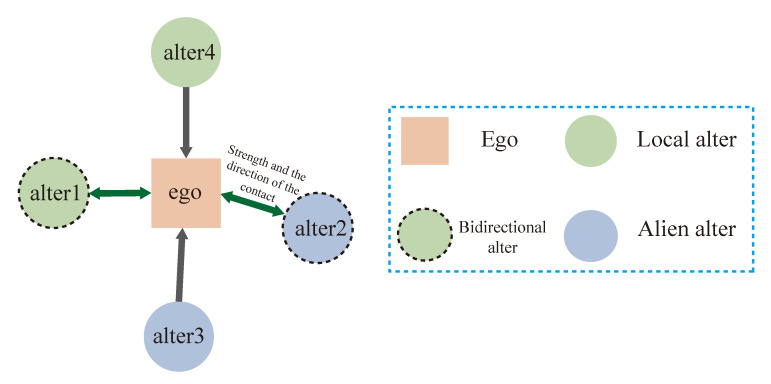
Data structure for social model based on egocentral network. The square box represents the ego and the color of the box indicates which group the ego belongs to. The color of the circle indicates whether they have something in common. The length of arrow tells us the relationship between ego and the alter. The color of an arrow represent the direction of contact. The dotted border indicates the alter is a bidirectional alter.

It is a common way to use graphs to represent social networks [33]. Both directed and undirected graphs are used in the research [4,42]. General speaking, bidirectional contact usually shows stronger intimacy than unidirectional [33] and is full of research value. Thus, in order to preserve the difference and information between bidirectional contacts and unidirectional contacts, we abstract the social network into a directed graph G(V,E), where *V* and *E* respectively represent the number of nodes and the number of links in the graph. $l_{i,j}^{t}$ is a link from *i* to *j* at start time *t*, and the weight $w_{i,j}^{t}$ means the value of contact between *i* and *j* from the start time *t*.While the methods to quantify contact are different in different social networks, it can be concluded that the contact between two people is measured by the total counts, the strength of each contact and the direction. Figure 1 shows our data model using the above concepts, and the longer the arrows are, the less intimate the relationship is. The dotted border indicates the alter is a bidirectional alter. The square box represents the ego and the color of the box indicates which group the ego belongs to. The color of nodes means that whether they have something in common. For telecommunication or email, it means whether they are from the same operator or service provider, while for Twitter and Weibo, it means whether they have joined in the same topic. Without special explanation, we will all represent the contact from ego to alter with contact-out, and from alter to ego with contact-in. The same to alters, we use alter-in and alter-out to represent alters who have contact-in or contact-out behavior, and we use local alter and alien alter to show whether the alter and the ego have something in common, such as interests.

### 4.2. Metrics Abstract

In the last section, we build a general model based on the ego central network for anomaly detection. In addition to it, abstracting metrics is another important thing. However, since the different egos may have different behaviors in different social communication methods, it requires us to deeply abstract the data, and extract the common metrics of the egos. After investigating various social networks, we designed the following metrics for analysis. For the ego *i* in the social network, we can divide its alters into two parts: $S_{in}^{i}$ and $S_{out}^{i}$, representing the alter-in set and the alter-out set respectively. $k_{in}^{i}=\left|S_{in}^{i}\right|$ and $k_{out}^{i}=\left|S_{out}^{i}\right|$ mean the in-degree and out-degree. They can characterize the influence of the ego, which means the role of ego in the whole network. For example, the ego with more out-degree indicates that he is more willing to maintain relationships, while the one with more in-degree shows that he is more attractive to alters [47], thus suggesting the ego’s size of the network. From the previous subsection, we can infer that the $w_{i,j}^{t}$ is important for us to measure the importance of alter *j* to ego *i*. Therefore, p

(1) $$W_{i}=\sum_{j\in S_{out}^{i}}\sum_{t}w_{i,j}^{t}$$

can quantify how ego *i* pays attention to his/her community. For normal egos, their total weight should not in an anomaly range. Contact-in and contact-out can help us judge whether the ego has a strong attraction and the importance of alters to egos, respectively. So we use the balance of attraction $\tau_{i}$:

(2) $$\tau_{i}=\frac{k_{in}^{i}}{k_{out}^{i}}$$

to measure the relationship between alters and egos. Among them, the closer the ratio is to 1, the more balanced the ego is, and the more stable the network structure is. The closer to 0, the greater the attraction of the ego, while the larger than 1, the weaker the ego’s attraction. In the last 2 cases, they all mean anomalous. From the previous section we know that the direction of contact is of great research value. Bidirectional alters are more likely to show intimacy than unidirectional alters, and if the ego’s alters are basically unidirectional, then he is very likely to be an anomalous user. We introduce relationship balance $\delta_{i}$:

(3) $$\delta_{i}=\frac{\left|S_{in}^{i}\cap S_{out}^{i}\right|}{\left|S_{in}^{i}\cup S_{out}^{i}\right|}$$

to measure the abnormal degree of the ego’s relationship. δ=1 means all the alters in the network are bidirectional, while δ=0 means all the alters are unidirectional. The proportion of bidirectional and unidirectional alters in the normal user’s network should be maintained within a normal range. In other words, too big or too small are both anomalous.

The temporal features of egos, such as posing/calling interval /frequency, can characterize their behavior habits, patterns and properties. Therefore, we use the time sequence vector $T_{i}$:

(4) $$T_{i}=\left\{t_{k}^{i},k=0,1\cdots ,23\right\}$$

to show the behavior of the ego *i*. $t_{k}^{i}$ means at time k the ego *i*’s features. As normal egos’ energy is limited, the reflect on the $T_{i}$ is that the time sequence of normal egos should be regular and have or resemble a shape of hump (having meals or break) and there should have none-active place (sleeping), which means there is no behavior during this period.

Above all, we propose the following metrics:

M1. Ego network’s in-degree $k_{in}^{i}$ and out-degree $k_{out}^{i}$.

M2. Ego network’s weight $W_{i}$.

M3. Attractiveness Balance $\tau_{i}$.

M4. Relationship Balance $\delta_{i}$.

M5. Time sequence vector $T_{i}$

These features can help us to make a preliminary classification of egos from a group perspective. The egos who unlike others can be found out. In order to analyze and judge these suspicious egos more deeply, we also need sociological methods to help make decisions. We will introduce them in next section.

### 4.3. Anomaly Detection

In anomaly detection, there is a dilemma that we have difficulty obtaining or only having a very small portion of the tag data. This makes it difficult for us to make a clear distinction between anomalies and normality. Besides, anomaly detection should be quick, but in social networks, the network shape is changing and growing at any time. These are the problems we need to solve. Finally, after careful consideration in our mind, we choose the local outlier factor (LOF) [48] model to solve the challenges. The reason why we choose it is beacuse: (1) Compared with supervised learning methods, it can be used directly without label dataset training and thus has a wider application range. (2) It can quantify the anomalies into scores instead of the labels, and let us find users who need to be explored in depth intuitively and efficiently. (3) It is a density-based detection method. Its detection results can be directly responded by dimension reduction.

An ego *p*’s anomalous score $LOF_{k}\left(p\right)$ based on his k-distance neighborhood is defined as followed:

(5) $$LOF_{k}\left(p\right)=\frac{\sum_{o\in N_{k}\left(p\right)\frac{lrd_{k}\left(o\right)}{lrd_{k}\left(p\right)}}}{\left|N_{k}\left(p\right)\right|}=\frac{\sum_{o\in N_{k}\left(p\right)}lrd_{k}\left(o\right)}{\left|N_{k}\left(p\right)\right|\cdot lrd_{k}\left(p\right)}$$

(6) $$reach-distance_{k}\left(p,o\right)=max\left\{d_{k}\left(o\right),d\left(p,o\right)\right\}$$

(7) $$d_{k}\left(p\right)=d\left(p,o\right)$$

(8) $$lrd_{k}\left(p\right)=1/\frac{\sum_{o\in N_{k}\left(p\right)}reach-dist_{k}\left(p,o\right)}{\left|N_{k}\left(p\right)\right|}$$

$d_{k}\left(p\right)$ is the k-distance of *p*. $reach-distance_{k}\left(p,o\right)$ is the k-reach-distance from node *o* to *p*. It represents the maximum value between the k-distance of *o* and the real distance between *p* and *o*. $N_{k}\left(p\right)$ is k-distance neighborhood of node *p*, which means it is a set include all the nodes that less than or equal to the k-distance of *p*. $lrd_{k}\left(p\right)$ is the local reach-ability density of node p based on $N_{k}\left(p\right)$. It means the local density of the current point and its surroundings. From the equation we can know that when $lrd_{k}\left(p\right)$ is higher, the *p* is more likely to be a normal node. Above all, we can conclude that if a node’s $LOF_{k}\left(p\right)$ is higher, it indicates the node is more different with its local k-neighbor. Generally, an ego i’s feature vector is shown below:

(9) $$f_{i}=\left[k_{in}^{i},k_{out}^{i},W_{i},\tau_{i},\delta_{i},T_{i}\right]$$

It can describe the global properties of the ego, which is important in anomaly detection. We use $F=\{f_{i},i\in G\}$, the collect of $f_{i}$ and the LOF to find out a preliminary detection results, and then do more in-depth research.

## 5. System Design and Overview

### 5.1. System Design

With the development of social communication, the data in it is getting larger and larger, which is very painful for experts to analyze and thus they want to have a convenient tool to do research. After our study, we find that multi-level analysis can help them make decisions, especially in unlabeled data. In addition to these, although there have been many studies aiming at detecting anomalies in social networks, they only focus on specific types of social networks, such as blogs, e-mail or telecommunications, and fail to make full use of the common patterns. Based on these requirements, we design a novel and general visualization system, egoDetect, which can be explored from macroscopic, mesoscopic and microscopic levels. The design requirements for these three levels are as follows:

Through the model in the previous chapter, we can select some suspicious users. While displaying these users, we should also show those users who are suspected to be normal, because the results of the algorithm cannot guarantee that they are all correct and we need a comparison to find out the difference between them. Therefore, the macroscopic level view’s aims are to display the whole picture of the network and help us make a preliminary classification of data.

- T1 Revealing Egos’ Features and Patterns. Since the model we use is multidimensional, we need a descending dimension to display the whole egos in the network. We should ensure that the relationship between egos is not lost in dimensionality reduction, which can help us to determine which points of the network are suspected egos.
- T2 Simple and Intuitive. The view should allow researchers to intuitively understand the inside of the network by using the anomaly detection’s results and though it, they can determine the node they want to study in depth is where.

Through the mesoscopic level view, the experts can select the ego they want to study deeply. Then, they need a more detailed view to help them make decisions. It is found that the relationship between two persons is important and a person’s relationship with others should be stratified [5,6,7]. In other words, everyone’s energy is limited, so people choose to spend a lot of time socializing with a small number of people and a small part of time communicating with others, but the hierarchical structure of abnormal users is generally vague. For advertisers, they are more likely to show an outward-spreading structure, that is, they like to contact other people but the relationship is not strong, while robot accounts show a high degree of intimacy with many people. Therefore, we need to reveal the relationship between the egos and alters in a mesoscopic level view.

- T3 Showing the Relationship Between Egos and Alters. As discussed above, network topology is very useful for exploring the ego. Therefore, we need to reveal the connection between egos and alters. In this way, experts can make a more in-depth analysis from a sociological perspective.
- T4 Drawing Egos’ Portrait. In addition to showing the relationship between egos and alters, we also need to build a user portrait based on these data to help experts understand the whole network.

The mesoscopic view is to analyze egos from a holistic perspective, so it does not provide more detailed information about egos. Anomalous egos not only differ in topology from others, but also in many other aspects, such as the behavior, patterns, active time and so on. Sometimes abnormal egos’ topology cannot be judged directly. Above all, the microscopic level view needs to provide the behavior, patterns and other detailed information.

- T5 Exploring Time Sequence. Normal egos should have a specific active time, which may vary according to their occupations, but people’s energy is limited, so it should have a hump shape and 0 valued region. Robot accounts and anomalous accounts will show long-term or even full-time behavior, while others may display local and random behaviors.
- T6 Analyzing Alters. The alters are those who egos contact-in or contact-out with. They form the networks of egos. Through the abnormal score of alters, we can judge egos from the other side. Besides, when we find an interesting alter from a mesoscopic perspective and want to dig deeper, or when we want to have a deeper understanding of the ego’s behavior with each alter, it can give us more information.

### 5.2. Solar Ego Network Model

In order to use the egocentric network to display the relationship between users and alters, we did a lot of studies and find that the traditional egocentric network view adopts node-link mode, where each edge represents the strength of the relationship between the ego and alter. However, this method does not intuitive enough, and with the increase of data, visual clutter is very serious. Therefore, we believe that a new view should be designed to clearly show the network structure and relationship with each contact in any situation.

Previous research indicates that all alters of an ego should have a hierarchy [6,7]. However, we find that anomalous users do not have this feature. Therefore, we decided to use this model to display the egocentric network and analyze egos by observing its network structure. However, here comes another question: What method should we use to determine the number of layers? There are already many researches and mature algorithms, such as Jenks Natural Breaks Classification [49] and Head/Tail method [50]. The main problem of them is that different people may have different network structures. Especially, anomaly egos have various structures, so it is hard to use algorithms to define uniform measurement indicators. Thus, we have come up with a compromise method to quantify each alter of ego according to the formula, and then map it to different layers in the graph according to the value. This ensures that different users can be properly displayed. Besides, previous studies have shown that most users’ alters can be divided into five categories, so our model uses a five-layer network structure.

**Figure 2:**
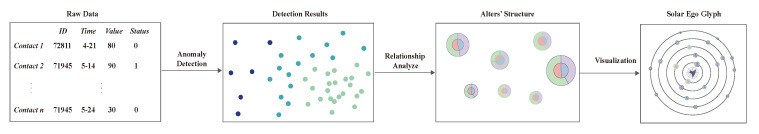
Workflow of the solar ego model.

Through the above research, and inspired by the solar system, we design a novel view to solve it. The pipeline of it is shown in Figure 2. It consists of a central node and five layers of tracks, the closer to the central node, the more intimate with egos. The distance $\theta_{i,j}$ represents the relationship between the ego *i* and the alter *j*, The equation of $\theta_{i,j}$ is as follows:

(10) $$k_{i,j}=\frac{max(C_{i,j},C_{j,i})}{min(C_{i,j},C_{j,i}))}$$

(11) $$\theta_{i,j}=k_{i,j}\cdot \frac{1}{\left(C_{i,j}+C_{j,i}\right)}$$

$C_{i,j}$ means the number of contact-out from *i* to *j*. From the equation, we can get that it is determined by the number of bidirectional contacts, so if the alter is unidirectional, the $k_{i,j}=-1$, which means the relationship between them is weak. With this glyph, experts can analyze more efficiently. With $k_{i,j}$, each alter can be placed on the corresponding layer.

### 5.3. System Overview

Motivated by the above requirements, we design the egoDetect to detect and analyze the anomalous users at three different scales: A group view to show the scatter of all egos through their features and anomaly scores, the topology and features in ego network with an ego view, and the more detail of the ego and between egos and alters showing in the detail view.

**Figure 3:**
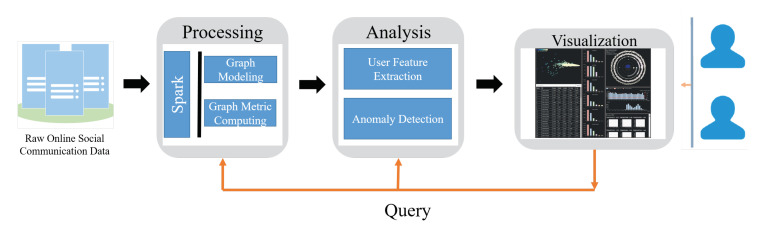
The data processing pipeline.

The data pipeline of the system is shown in Figure 3. The raw data storage in HDFS (Hadoop Distributed File System). We use Spark to model graph and compute the metrics of it, and use the results to build egos’ features. With the features, we can get the anomaly detection score of each ego in the network. Then, we use multidimensional scaling (MDS) [45] to reveal the scatter of them and use a novel glyph to show the ego network of them. We design our view through the D3 and Echarts, using Flask as our framework.

## 6. Visualization

The goal of our system is to assist experts in identify and verify anomalies of users in social networks which are lack of unlabelled data, and provide multiple views to validate. We also offer a lot of interactions to help researchers. In this section, based on the design tasks and requirements outlined in the previous sections, we will give a detailed description of the system and each view in the system.

### 6.1. User Interface

**Figure 4:**
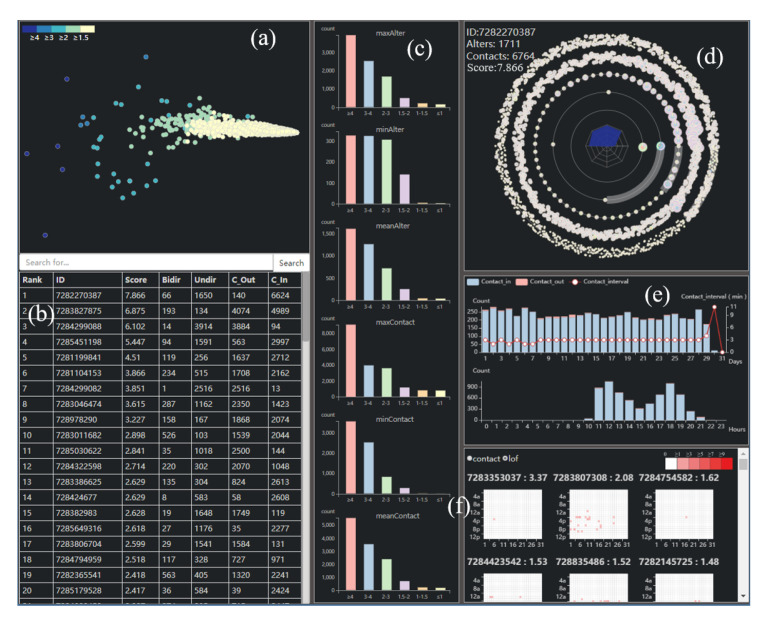
The overview of egoDetect based on the call record data. The user interface consists of six parts: (**a**) The distribution of users with their features, (**b**) a list sorted by users’ anomaly scores, (**c**) statistical information for each segment, (**d**) the ego network glyph inspired from solar system, (**e**) the statistical view of ego’s active time and behavior, (**f**) the detail view about the contact between the ego with each alter.

Guided by the above requirements, our system is designed as Figure 4. The interface consists of six major UI components: (a) a view mapping user’s multi-dimensional features into two-dimensional space and showing in the feature space; (b) a list of whole users’ detailed features sorted based on LOF anomaly scores; (c) some statistical information for each segment; (d) a novel ego network glyph inspired by the solar system for visualizing the structure of the ego’s network and the relationship between alters and egos; (e) a statistical view representing the active time and habit of the ego; (f) a detail view to display the contact between ego and each alter and the anomaly of each alters. In summary, (a), (b), (c) compose our group view, (d) is our ego view, and (e) and (f) are our detail view. We will introduce the design of them as follows.

### 6.2. Group View

The group view is aimed at displaying the whole topology of the network and helping us make a preliminary classification of data. Multidimensional scaling (MDS) reducts based on the distance between points, so if the points are more similar, the closer they are after dimension reduction. With the using of it, we can reveal the distribution of each ego by their features and find out whether the algorithm results are effective (T1). The result is shown in Figure 4a, and in order to distinguish anomaly scores of each ego, we color code their score into five colors, representing their anomaly degree (T2). Scatter plot can help experts to have a detailed understanding of the internal situation of the social network. However, due to dimensionality reduction, on the one hand, it is impossible for experts to have an understanding of egos through x and y coordinates, so we need to provide the specific characteristics of each ego to help them make decisions. As shown in Figure 4b, we design a list based on the ranking of LOF scores to show the detail of each ego. On the other hand, the MDS dimension reduction brings similar points together. With the increase of data, visual clutter becomes more serious and we cannot understand the overall situation of the network, so we provide statistical data based on the detection results. After experiments, we find that the score of users below 1.5 are more than 90%, so we take 1.5 as the boundary and classify the users into 6 groups. We select the maximum, minimum and average values of alters and contact times for each segment. These allow us to have an intuitive understanding of each fraction. Above all, we design a statistical view to help experts analyze. (T2). The design is shown in Figure 4c.

### 6.3. Ego View

Through the analysis of the group view, experts can pick out the points they want to continue analyze. In this view, we need to display the relationship between the ego and all his/her alters, so that experts can explore through a sociological perspective. Figure 4d is the ego view of our system. It consists of two parts: An ego network glyph based on solar system and an ego’s portrait.

**Figure 5:**
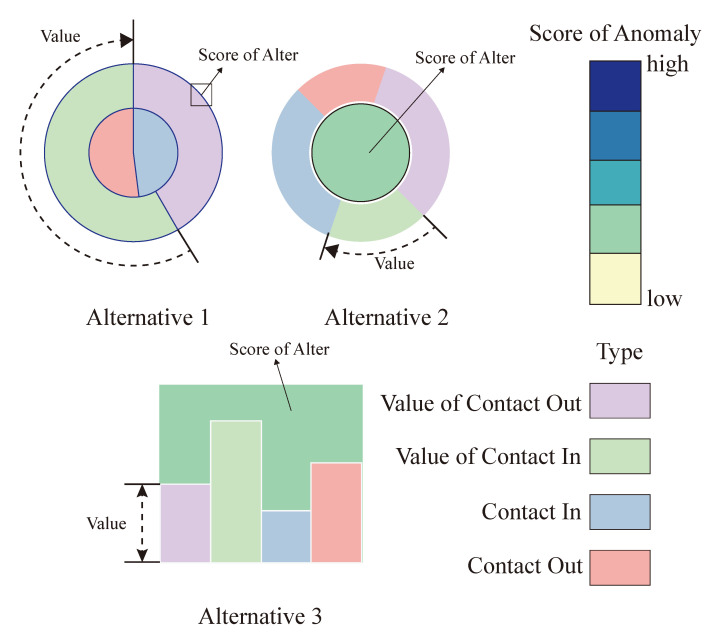
Design alternatives of alters.

Ego Network Glyph. As mentioned above, we design a novel glyph to reveal the patterns between egos and alters. Figure 4d is our solar network glyph. Each alter is a node surrounding by the ego. The distance between the ego and an alter and the radius of the node represents the relationship between them. The grey transparent ring in each track tells us the proportion of local alters’ number to the total number in this track, and the nodes in it mean they are the local alters, while the nodes not in it mean the alien alters. Above all, it can give us an intuitive and detailed understanding of the internal structure and circumstances of an ego network (T3). It is worth noting that how to code alters, as an important part of the network, is a very important issue. We consider three alternatives to visual encodings of each alter, showing in Figure 5. In the first design, the inner ring shows the count of contact-in and the contact-out, like the count of contacts in Twitter, while the outer ring is the value of the inner ring, such as time spending on calls. We also use the line color to show the anomaly score of the alter. However, when the circle is too small to see, it is hard to get the color of the line. The second design uses the inner and outer circle radii to encode the information of alters, and the center circle represents the anomaly score of them. When the alters become large, we find it is sometimes confused about the color coding. The third design is based on the bar chart, where each column represents one information of the alter. It is limited by shape. When the amount of data is large, the data clutter is serious. While all three designs have some drawbacks, we finally used the first design after careful consideration. Because there are egos contact with many alters in social networks, we need to make sure that they are still as clear as possible when they are presented (T3).

**Figure 6:**
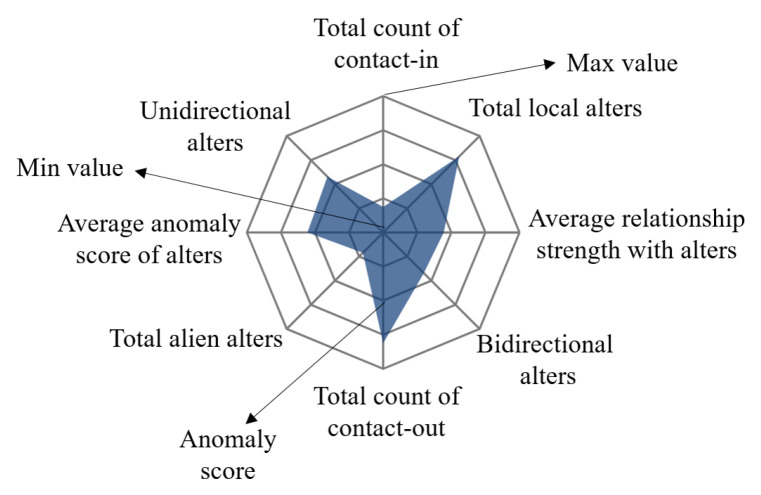
Ego features radar view with six dimensions.

Portrait Glyph. Through the contact data of ego, we can conclude the various characteristics of the ego and they can help us judge whether the ego is abnormal. In addition to some common indicators, like total count of contact-in and contact-out, we introduce average relationship strength with alters, average anomaly score of alters, local alters and alien alters, unidirectional and bidirectional alters features into the system. The average relationship strength with alters helps us analyze ego’s behavior patterns. Many anomalous egos are machine users generated by software, so there will be many similar behavior patterns and like to contact with each other. Besides, for advertisers, they tend to contact-out more than contact-in and have little bidirectional alters, leading to a low level of average relationship strength with alters, which can respond from these attributes. The local alters, alien alters and unidirectional and bidirectional alters properties help us to understand the egos’ alters’ structure. Normal egos are more likely to contact people who have similar interests and hobbies with them and the proportion of unidirectional and bidirectional alters should be balanced. The average anomaly score of alters can tell us how the average anomaly of alters is. As mentioned above, machine users will contact other machine users. If an ego’s alters’ anomaly is high, the ego can also be considered an anomalous user. In the solar network layout, the ego’s information is located in the middle of the whole network (T4). In order to better display ego’s data from multiple dimensions, we choose the radar map as the center of the whole layout. All features of the user are mapped to each direction of the radar map, and the center of each direction means the minimum while the outside means the maximum. The color of the area represents the anomaly score of the ego. As shown in Figure 6, radar maps can simply and directly highlight important information.

### 6.4. Detail View

It is often not enough to only provide the overall information of an ego. Sometimes anomalous egos may have no difference with others in a holistic perspective, or we want to dig deeper about egos’ behavior with alters, and thus we need more detail. Based on these, the design of a detail view is shown as Figure 4e,f. Figure 4e is a statistical view showing the time sequence information about the ego and Figure 4f represents the detail contact between the ego and each alters.

Statistical View. In addition to exploring the topology of alters and egos, time series information about the ego is also one of the most important means of analysis. It can reflect egos’ social habits and behavior patterns, and is also a part of the common criterion in anomalous analysis. The active time of the ego can reflect his habit, and tell us his general lifestyle. The value of his contact-in and contact-out represents the structure of his contact. Normal users should have a regular or relatively regular active time, as well as a normal structure and number of contact. In addition, the ego’s daily contact interval is also very important to measure whether he/she is normal. Experience shows that there is no regularity in the contact interval of normal people. Thus, the statistical glyph is designed as Figure 4e. We use a polygon and a histogram to display time sequence data, making it easier for us to know the ego’s active time and habit (T5).

**Figure 7:**
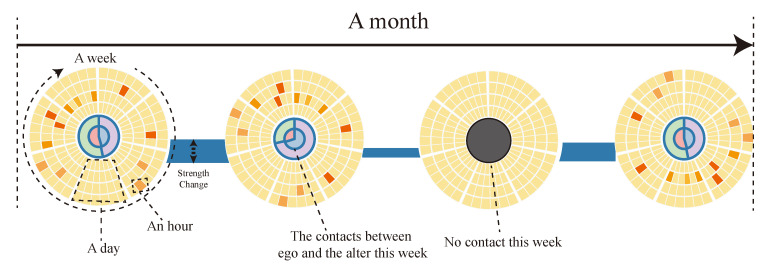
An alternative design for detail view based on circular layout.

Detail View. The contact between the egos and alters may occur at any time of the day, if we display the full time series of every day, it is very difficult to visualize in a small space, so we make a compromise, using the heat map to display the contact between the ego and each alter. The heat map shows the size of the data values in a highlighted way and can intuitively show local anomalies. In the heat map, the horizontal axis represents the time period, the vertical axis represents the hours during a day, and the color of each grid represents the number of times that the ego and the alter communicate with each other during that period. The title of each view consists of alter ID and its anomaly score. We combine the heat map with the timing information to show the ego’s contact timing diagram with each alter (T6). Besides, in order to explore the anomalies of the ego from the alters, the detail view can be sort by the anomaly score from high to low or by the strength with ego and visualize them (T6). It is shown in Figure 4f. We also experiment with another design, using a circular layout. In Figure 7, each sector represents a day, and a lattice in the sector shows a period of time. However, we do not use this because the utilization of circular layout space is not high, and it is difficult to make horizontal and vertical comparisons.

### 6.5. User Interactions

We add some interactions in the system to help the researchers to use the system more smoothly.

Filter. In order to help the experts have an understanding of the users of different score segments in the network, we provide filtering interaction through the color bar in the Figure 4a.

Search. The search interaction is provided by the search bar in Figure 4b. The researchers can search for the ego they find interesting in the Figure 4a.

Highlight. Most of the elements in the system can be highlighted. For example, when the mouse is over one line in the Figure 4b, the node of this line can be highlighted in the Figure 4a synchronously. When the mouse is over the alters in the Figure 4d, the location of it can be found and highlighted in the Figure 4f.

Zoom In and Out. The view in the Figure 4a can be zoomed in and out for the detail.

Click. If we find an ego in the Figure 4a,b or an alter in the Figure 4d we want to know more, we can click on the line or the circle, then the ego view or the detail view will be drawn.

Tips. Our system can give you some tips when you are confused or forget something.

## 7. Case Study

In this section, we will apply our system in the task of anomaly detection with a call record data provided by an operator to demonstrate the effectiveness of our system.

In this study, the dataset is provided by one of the largest mobile operators in China. It covers 7 million people of a Chinese provincial capital city for half a year spanning from January to June 2014. According to the operator, all the users can be divided into two categories the local users (customers of the mobile operator who provide this dataset) and the alien users (customers from the other operators). The reason for such distinction is that the communication behaviors of alien users are not recorded and cannot be collected by our data provider based on policies. Therefore, we have to put our focus on the local users whose entire calling behaviors are recorded within the dataset. We won’t show the alien alters’ details, such as the score of them in the detail view and ego view. In order to protect the user’s personal information, we encrypt all telephone numbers and embody the characteristics of local and alien directly on the user ID. So 728 indicates local user, and 719 indicates alien user. Each user has his own unique ID. The basic statistics of the mobile communication data are summarized in Table 1.

Parameter Selection. As described in Chapter 4, for the LOF algorithm, the most important parameter is its neighbor number n. We find that the n has a great influence on the score of egos in sparse and dense boundaries. In order to ensure that the points in these areas can be classified more accurately, we need to compare them with enough points, so we select a larger n, which can ensure that the points in dense or sparse areas do not have an impact, but can give more full consideration to the points in the boundary. In feature selection, we find that if we do not consider the time sequence, some points with anomalous behavior cannot be detected. We found that because their attributes, such as the number of alters, the number of calls and so on, are not different from normal people, leading to the failure to detect. However, when we introduce the time sequence, the problem is improved.

Exploratory Analysis. First of all, we have made a preliminary exploration to get the whole picture of the entire dataset. From the group view’s MDS map, shown in Figure 4a and containing all the ego information, we can find that most of the points inside the network are concentrated together, and only a small number of points are distributed in the periphery with high anomaly scores. This shows that most users are regarded as normal users when they gather together, while a few users are regarded as outliers when they are distributed at the edge. When we zoom in on the view for observation, we find that egos with higher scores also appear in places with lower scores, where is a place worth studying.

From the list in Figure 4b, we notice that nine egos with more than three points, and from the Figure 4c, the data of each segment shows a downward trend. We also find that the number of alters with anomaly scores less than 1 does not exceed 150, but they make up only about 6% of the population, while the percentage of egos whose anomaly scores are less than 1.5 is about 95%, and the alters’ number of them is no more than 216, which is larger than the Dunbar’s Number. We think the reason for this is that communication is bidirectional firstly, which means that you may receive calls you don’t want to answer, leading to an increase in the number of contacts. Secondly, it may be affected by the algorithm, and there is the possibility of misclassification. We need a deep analysis to validate.

**Figure 8:**
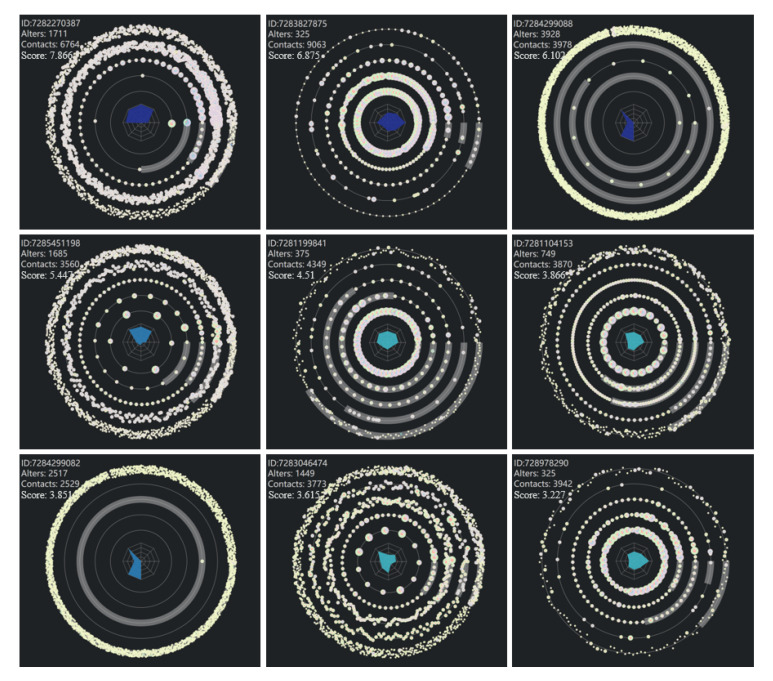
Top nine users’ ego views. Decrease from left to right and from top to bottom.

**Figure 9:**
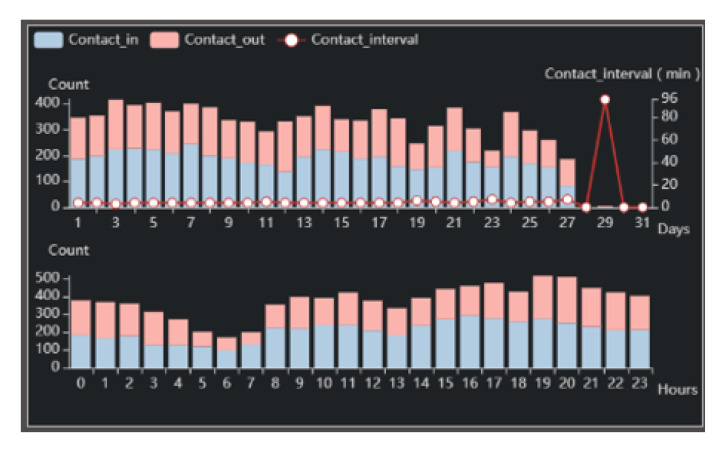
Second ego’s statistical view.

Results. In order to further verify the effectiveness of the system, we proceed from the actual case and demonstrate the system. We have drawn ego views of all users with ratings greater than 3. As shown in Figure 8, their alters and calls are particularly numerous. They mainly show two kinds of structure, either focusing on the outer layer, showing the characteristics of advertisement users, or mainly focusing on the inner layer, showing the characteristics of robots. From their central radar maps, we can see that they have distinct convex shapes. Figure 4f is the first ego’s detail view. We find that this one contacts many users with higher anomaly scores and contacts in more than contacts out, through understanding, we find that this is a customer service of the scam group. For the second ego, shown in Figure 9, we find that he is a highly active user with average contact interval, so we think he is a robot account. Those users who scores more than three points can initially identify anomalous users from the group view, which is further confirmed by the analysis of ego view.

**Figure 10:**
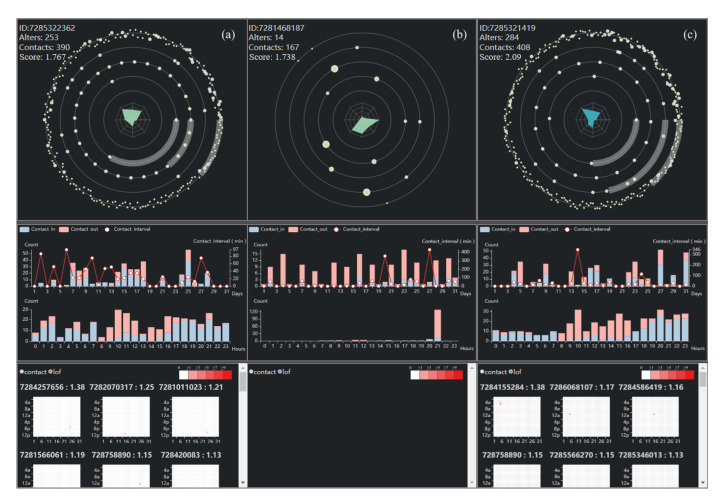
Some examples; (**a**) is the ego who cannot be judged directly by ego view, (**b**) is the ego who is misclassified, (**c**) is the ego who intends to disguise as a noraml one, but actually is an anomlous ego.

At the same time, some egos cannot directly judge whether they are anomalous users through group view, such as ego: 7285322362, shown in Figure 10a, whose alters and calls are not high, but from his solar ego glyph we can find that his connection with alters is weak, and from his detail view we can also find that his active time is different from normal people, so he is an abnormal ego. As mentioned above, there are some nodes with high anomaly scores in an area full of low scores. We speculate that they may be normal egos with abnormal behavior or abnormal egos disguised as normal egos. So we have a detailed analysis of these points. Like ego: 7281468187, shown in Figure 10b, his score is 1.738, and can be considered as an anomaly ego. However, after our exploration, we find that although the behavior pattern is slightly different from that of ordinary people, the network structure of his contacts is not abnormal, so we think that this is a normal ego who shows abnormal behavior. While the above ego has high anomaly scores, further analysis shows that he is not really abnormal, just because he behaves differently from normal egos. In the dense areas, we believe that there are also really anomaly egos. As shown in Figure 10c, it is an anomalous ego that we have found. While we can’t tell if he’s abnormal from his solar ego glyph, when we go deep into his behavior patterns, we find that he has signs of full-time activity, so we conclude that he’s an abnormal user. We think that this kind of nodes is anomalous egos who want to mix up with normal egos and disguise as normal egos, but they can still be detected by our system. After communicating with experts, they confirm that our speculate is correct and show that our research is very helpful for them to mine potential abnormal users.

We have fully investigated the anomaly detection results by our system and many interesting patterns are found. We also invite several experts from our data provider and telecom data analysis field to help us understand and check our findings. First, we show them the abnormal users with high scores, as shown in Figure 8. They share the same idea with us. For example, the first ego, ID:7282270387, is a customer service of the scam group, while the second user, ID:7283827875, is a robot account. Next, we verify some controversial nodes, like nodes with high scores in an area full of low scores, with them. They all feel that our discovery is valuable and prove that our discovery is useful. They verify that, for example, ego:7285321419, shown in Figure 10c, is indeed a potential abnormal user. However, the results involve the privacy of users, so they do not disclose more detail to us.

## 8. Discussion

The egoDetect, a method proposed in this paper, mainly uses visualization to capture and analyze the anomalies in online social network from the perspective of ego network. The analysis of communication data has been verified by experts, which proves the effectiveness and usefulness of the method. When designing the corresponding visual coding and layout algorithm, scalability is our special concern. Our method starts from the macroscopic level, looks for suspicious anomalies by comparing the status of all nodes in the network. Then, it analyzes suspicious objects by ego network from the mesoscopic level. Finally, from the microscopic level, it analyzes suspicious objects using time series data. In other words, through the high-level abstraction and generalization of the objects, we ensure the scalability of the method. Based on this, we can focus more on the objects themselves, rather than how many attributes there are and what each attribute means. Therefore, this method has good scalability and generality, and can perform well in different fields of time series data. For example, in the field of IOT and cyber-physical social systems, sensors, the basis for data collection, are the most critical part. If the sensors function abnormally or are attacked maliciously, the validity of the data cannot be guaranteed, and the processing and analysis cannot be carried out. Therefore, it is very important to detect abnormal sensors. Just as people can be compared to sensors, and vice versa. Besides, the data collected by sensors can be compared to the call data of people. Through anomaly detection and visual analysis of all sensors, we can quickly target dubious sensors. Then we can analyze dubious sensors based on the visualization of ego network and time series data, and finally we can find out abnormal sensors to ensure the security of the whole network and systems. Therefore, our method has strong scalability, and has great value for sensor networks, cyber-physical social systems and IOT.

## 9. Conclusions

In this paper, we propose a novel visualization system, which has novel visual glyphs and uses multi-view to explore, detect and analyze the anomaly in the social network. The system analyzes from macroscopic, mesoscopic and microscopic perspectives. We show the abnormal situation in the online social communication network after anomaly detection from a macroscopic point of view; in the mesoscopic view, we introduce galaxy maps, combined with the ego central network analysis method, to display the interested users in multi-dimensional, from the network structure, active time, alters intimacy and other aspects to judge the abnormal degree of users; and through the microscopic view, combined with timing, we can evaluate the abnormal degree of users from the point of view of alters. We also add friendly and intuitive interactions to help researchers quickly get the information they want. We use a call record data to demonstrate the system is beneficial for detecting abnormal behavior in online social communication. We also discuss the feasibility of applying the method to other fields, like IOT and cyber-physical social systems.

However, limited by time and energy, our work still has a lot of room to improve. Through the case study, we find that although the LOF algorithm can help us to mine latent anomalous egos by combining time series, it also incorrectly classifies some normal errors. Restricted by datasets, it is difficult for us to analyze alien alters and egos, which is disadvantageous to our analysis.

In the future, we plan to design better anomaly detection algorithms. This can make our detection accuracy higher. Besides, the dataset used in this experiment is only provided by a certain operator, so there are limitations in the analysis of specific contacts. In the follow-up experiments, we hope to deepen cooperation with other operators, obtain more and more communication data from the external network, and conduct more in-depth research.

## Figures and Tables

**Table 1:** Basic statistics of the mobile communication networks.

Time	$N_{t}$ (*Total* Users)	$N_{l}$ (*Local* Users)	$L_{t}$ (*Total* Links)
Jan.	6520121	751643	32521180
Feb.	6234877	742504	27600221
Mar.	6481767	783751	32720452
Apr.	6526250	777486	32383231
May	6561107	787614	34119390
Jun.	6531076	787156	33461297
